# Identifying the ERAD ubiquitin E3 ligases for viral and cellular targeting of MHC class I

**DOI:** 10.1016/j.molimm.2015.07.005

**Published:** 2015-12

**Authors:** D.J.H. van den Boomen, P.J. Lehner

**Affiliations:** Cambridge Institute for Medical Research, Department of Medicine, University of Cambridge, Cambridge CB2 0XY, UK

**Keywords:** ER-associated degradation, E3 ubiquitin ligase, Human cytomegalovirus, Viral immune evasion, TRC8, TMEM129, HRD1

## Abstract

The human cytomegalovirus (HCMV) US2 and US11 gene products hijack mammalian ER-associated degradation (ERAD) to induce rapid degradation of major histocompatibility class I (MHC-I) molecules. The rate-limiting step in this pathway is thought to be the polyubiquitination of MHC-I by distinct host ERAD E3 ubiquitin ligases. TRC8 was identified as the ligase responsible for US2-mediated MHC-I degradation and shown to be required for the cleavage-dependent degradation of some tail-anchored proteins. In addition to MHC-I, plasma membrane profiling identified further immune receptors, which are also substrates for the US2/TRC8 complex. These include at least six α integrins, the coagulation factor thrombomodulin and the NK cell ligand CD112. US2’s use of specific HCMV-encoded adaptors makes it an adaptable viral degradation hub. US11-mediated degradation is MHC-I-specific and genetic screens have identified TMEM129, an uncharacterised RING-C2 E3 ligase, as responsible for US11-mediated degradation. In a unique auto-regulatory loop, US11 readily responds to changes in cellular expression of MHC-I. Free US11 either rebinds more MHC-I or is itself degraded by the HRD1/SEL1L E3 ligase complex. While virally encoded US2 and US11 appropriate mammalian ERAD, the MHC-I complex also undergoes stringent cellular quality control and misfolded MHC-I is degraded by the HRD1/SEL1L complex. We discuss the identification and central role of E3 ubiquitin ligases in ER quality control and viral degradation of the MHC-I chain.

## Highlights

• E3 ubiquitin ligases play a central role in viral and cellular degradation of MHC-I.
• HCMV US2 and US11 hijack the mammalian ERAD machinery to induce MHC-I degradation.
• We identified the TRC8 and TMEM129 E3 ligases as crucial for US2/11 function.
• The US2/11 degradation hubs are flexible and enable viral evasion of different immune functions.
• Cellular quality control of MHC-I is controlled by the HRD1/SEL1L E3 ligase complex.

## 1 Introduction

The essential role of the major histocompatibility class I (MHC-I) antigen presentation pathway in immune detection of intracellular pathogens is emphasised by the different strategies used by many viruses to interrupt this pathway (Hansen and Bouvier, 2009, Randow and Lehner, 2009). The ongoing battle between viruses and the immune system has resulted in many viruses co-evolving one or more gene products which inhibit MHC-I presentation, with the presumed aim of preventing viral peptide presentation by MHC-I to cytotoxic T-lymphocytes (CTL). This is exemplified in the herpesvirus family, with human cytomegalovirus (HCMV) providing one of the major paradigms for viral immune evasion. As the largest known human herpesvirus, HCMV encodes ∼170 canonical open reading frames of which only 45 are required for viral replication, with many of the remaining genes involved in immune evasion. The downside of this extensive range of viral gene products is that they provide a smorgasbord of potential viral epitopes for MHC-I presentation. In response, HCMV encodes at least four gene products that interfere with classical MHC-I antigen presentation (Hansen and Bouvier, 2009, Loureiro and Ploegh, 2006). HCMV US2 and US11 promote MHC-I reverse translocation from the ER to the cytosol for subsequent proteasomal degradation, US6 blocks TAP-dependent peptide transport, while US3 inhibits tapasin-dependent peptide loading and retains MHC-I in the ER. Here we review our recent findings on the central role of ubiquitination and the identification of independent host ubiquitin E3 ligases used for both viral and cellular regulation of MHC-I assembly within the ER.

## 2 US2 and US11 hijack mammalian ER-associated degradation (ERAD) to target MHC-I for proteasomal degradation in the cytosol

US2 and US11 are ER-resident type I membrane viral glycoproteins expressed early in HCMV infection. Upon co-translational insertion into the ER, newly synthesised MHC-I is immediately bound by US2 or US11 which induces its rapid degradation with a half-life of 1–10 min. The ER is a protein-folding environment and is mainly devoid of proteases or an ubiquitin-proteasome system. In a process now known as retrotranslocation or dislocation, US2 and US11 were shown to induce the reverse translocation of MHC-I from the ER back to the cytosol where the MHC-I is deglycosylated by protein *N*-glycanase (PNGase) and degraded by the proteasome (Wiertz et al., 1996a, Wiertz et al., 1996b).

Although retrotranslocation was first described in the context of US2/11-mediated MHC-I degradation, it turns out to be part of a common cellular pathway used for the degradation of misfolded secretory proteins from the ER—the so-called ‘ER-associated degradation (ERAD)’ pathway (Olzmann et al., 2013). The ER provides a folding environment for newly synthesised proteins and only those proteins that acquire their native conformation and pass the ER quality control (ERQC) checkpoint are allowed to exit the ER and traffic through the secretory pathway (Hegde and Ploegh, 2010). ERAD therefore provides a mechanism by which misfolded proteins can be safely removed from the ER by retrotranslocation back to the cytosol and degraded by the proteasome. US2 and US11 hijack this cellular quality control pathway and ‘force-feed’ it with folding-competent MHC-I to induce its rapid proteasomal degradation. As biological tools, US2 and US11 have been invaluable for the functional characterisation of mammalian ERAD pathways, and allowed constructive comparisons with related degradation pathways in yeast. ERAD is not just responsible for the degradation of misfolded proteins, but also plays a critical role in the regulated turn-over of ER-resident proteins, such as the activated IP_3_ receptor and the HMG-CoA reductase, squalene monooxygenase and heme oxygenase-1 (HO-1) enzymes.

## 3 A central role for E3 ubiquitin ligases in the ERAD pathway

Ubiquitination is central to ERAD and membrane-bound ERAD E3 ubiquitin ligases form the core of the retrotranslocation machinery. These ligases are surrounded by numerous ERAD factors required for substrate recognition, dislocation and membrane extraction. Misfolded protein substrates are recognized in the ER by a series of chaperones including OS-9, XTP-3B, EDEM1-3 and BiP and are recruited to the ligase via membrane components such as SEL1L and HERP. The substrate is then ubiquitinated by the ERAD E3 ligase and dislocated into the cytosol with the cytosolic AAA ATPase p97/VCP supplying the energy required for dislocation and membrane extraction. p97 is recruited to the ERAD machinery by membrane components including Derlin-1 and UBXD8. Although the exact nature and identity of the retrotranslocation channel remains unclear, the membrane-bound ERAD E3 ligase and components like Derlin-1 might form part of the retrotranslocon. The dislocated cytosolic substrate is finally deglycosylated by p97-associated PNGase and routed to the proteasome for degradation.

Despite the absolute requirement for ubiquitination in the proteasome-mediated degradation of ERAD substrates, its exact role in dislocation remains unclear. Depletion of the cognate E3 ligase with the resulting loss of ubiquitination inhibits dislocation of many ERAD substrates. However, as ubiquitination only occurs on the cytosolic side of the ER, partial retrotranslocation must necessarily precede ubiquitination for luminal ERAD substrates. Membrane extraction might again be ubiquitin-dependent as the AAA ATPase p97 and its co-factors Ufd1/Npl4 are thought to recognize substrates by their ubiquitin moiety. Yeast contain a minimal ERAD machinery with only three E3 ligases: Hrd1p which is thought to target proteins with a luminal and membrane folding defect (ERAD-L/-M); Doa10p which targets proteins with a cytosolic defect (ERAD-C); and the Asi complex which targets proteins of the nuclear envelope. The evolutionary diversification from yeast to higher eukaryotes greatly expanded the repertoire of ERAD E3 ligases which now include the Hrd1p homologues HRD1 and Gp78, the Doa10p homologue MARCH6/TEB4, RNF5/RMA1, RNF170, as well as TRC8 and TMEM129 (discussed below) and the cytosolic E3 ligase CHIP. The driving force for this diversity and the requirement for an increased number of ligases is not well understood, but increased complexity of the secretory system and cell–cell communication might underlie this phenomenon. US2/11 illustrate the increased complexity of the mammalian ERAD system. They both hijack the mammalian ERAD machinery to induce MHC-I degradation, yet these viral gene products function independently and co-opt distinct ERAD pathways. Despite their early role in defining mammalian ERAD, the host E3 ligases hijacked by HCMV US2 and US11 have only recently been identified.

## 4 The TRC8 E3 ligase ubiquitinates MHC-I and is rate-limiting in US2-mediated degradation

**Fig. 1:**
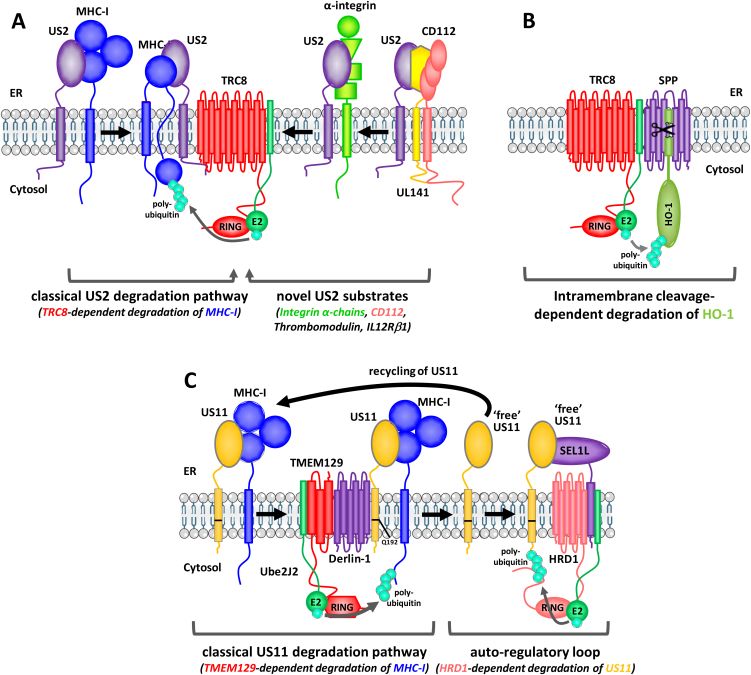
The US2 and US11 viral degradation hubs and their cognate host ERAD E3 ubiquitin ligases. (A) The US2/TRC8 degradation hub. US2 recruits the TRC8 E3 ligase via its cytosolic C-terminus. TRC8-dependent polyubiquitination of MHC-I leads to rapid retrotranslocation and degradation of the MHC-I heavy chain. In addition to MHC-I, the US2/TRC8 degradation hub degrades a wide variety of immune receptors including at least 6 different integrin α-chains, the anti-coagulation factor thrombomodulin, the IL-12 receptor β1 chain and the NK cell ligand CD112. Whereas US2/TRC8 is sufficient for the degradation of most substrates, efficient degradation of CD112 requires help from the HCMV-encoded ‘holdase’ UL141. US2 function is independent of SPP. (B) The TRC8/SPP degradation hub mediates cleavage dependent degradation of heme oxygenase-1 (HO-1). SPP-mediated cleavage of the HO-1 transmembrane domain releases HO-1 from the membrane and induces TRC8-dependent ubiquitination and proteasomal degradation. This pathway is used by other tail-anchored proteins. (C) The US11/TMEM129 degradation hub. US11-bound MHC-I is rapid ubiquitinated by the TMEM129 E3 ligase and subsequently retrotranslocated to the cytosol for proteasomal degradation. TMEM129 is recruited to US11 via Derlin-1. US11 is not itself a substrate for TMEM129, but is either recycled to bind another MHC-I molecule, or is degraded by the HRD1/SEL1L ERAD complex in an auto-regulation loop. In this way, HRD1-mediated degradation of ‘free US11’ buffers US11 levels to the MHC-I client load.

To identify the E3 ligase required for US2-mediated degradation, we used a flow cytometry based siRNA screen in US2-expressing HeLa cells using GFP-tagged MHC-I as the optical read out. Of the 373 predicted E3 ubiquitin ligases tested, depletion of only the TRC8 (RNF139) E3 ligase rescued MHC-I rescue from US2-mediated degradation (Stagg et al., 2009). TRC8 encodes an ER resident polytopic RING E3 ligase with a C-terminal RING-H2 and a unique N-terminal sterol sensing domain (SDD) and was previously identified as a result of a 3;8 translocation associated with familial renal clear cell carcinoma. TRC8 binds the cytoplasmic tail of US2 resulting in rapid MHC-I polyubiquitination, which triggers full retrotranslocation into the cytosol and subsequent proteasomal degradation of the MHC-I (Hsu et al., 2015, Stagg et al., 2009) (Fig 1A). Although US2-induced degradation is lysine dependent, lysines in the cytoplasmic tail of MHC-I are dispensable, suggesting TRC8 ubiquitinates the luminal domain of the MHC-I heavy chain (Furman et al., 2003). Partial dislocation would therefore precede ubiquitination, as seen in the dislocation of misassembled MHC-I in the absence of US2/11 (Burr et al., 2013). Interestingly, following TRC8 depletion, the presence of US2 is insufficient to retain MHC-I in the ER which escapes to the cell surface, suggesting the US2-MHC interaction is transient and unable to retain MHC-I in the ER in the absence of degradation (Stagg et al., 2009).

A key TRC8 interaction partner is the intramembrane cleaving aspartyl protease, signal peptide peptidase (SPP). Although SPP was suggested to be essential for US2-mediated degradation (Loureiro et al., 2006), neither a traditional homologous recombination-mediated somatic cell knock-out of SPP nor CRISPR-mediated SPP deletions in different cell lines affected US2-mediated MHC-I degradation (Boname et al., 2014). Furthermore, the absence of SPP did not affect the US2-TRC8 interaction. SPP does not therefore appear to be required for the US2-mediated dislocation of MHC-I. However, the finding of an intramembrane cleaving protease associated with an ERAD E3 ligase does provide a potentially attractive mechanism for ER protein turnover. In a subsequent proteomics study we found the tail anchored (TA) protein HO-1 accumulates in the absence of SPP (Boname et al., 2014). Tail anchored proteins are inserted into the ER membrane via a C-terminal transmembrane domain and adopt a type II membrane orientation, making them ideal substrates for SPP which only cleaves membrane proteins with a type II orientation, a conserved feature of all known SPP substrates. The HO-1 transmembrane domain is indeed cleaved by SPP thereby releasing it for TRC8 dependent proteasomal degradation (Fig 1B) (Boname et al., 2014). This mechanism of degradation is not unique to HO-1 but shared by a selection of other TA proteins. In the absence of an extended luminal domain, the release and degradation of these proteins might proceed without the need of a retrotranslocation channel with ubiquitination sufficient to drive their post-cleavage proteasomal degradation. Intramembrane cleavage by SPP or its cousin RHBDL4 might also aid in the degradation of proteins with a more complex membrane orientation including the transcription factor XBP1u, though their mode of extraction remains unclear (Chen et al., 2014, Fleig et al., 2012).

TRC8 function likely extends beyond the degradation of TA proteins. Its sterol sensing domain suggests an involvement in membrane lipid homeostasis, though the role of TRC8 in the processing of SREBP transcription factors and sterol-induced degradation of HMG-CoA reductase remains controversial (Irisawa et al., 2009, Jo et al., 2011, Lee et al., 2010, Tsai et al., 2012). The study of US2 has therefore uncovered a novel branch of mammalian ERAD with an E3 ligase involved in channel-independent degradation and lipid homeostasis.

## 5 TMEM129, a non-classical RING E3 ligase, mediates HCMV US11-induced MHC-I ubiquitination

A requirement for ubiquitin for US11 activity was demonstrated in a temperature sensitive E1 ubiquitin activating enzyme cell line but the role of substrate ubiquitination was confusing as US11 can target a lysine-less MHC-I molecule (Hassink et al., 2006, Kikkert et al., 2001). The recognition that ubiquitination can occur on non-lysine residues, including serine/threonine and cysteine, provided a potential solution to this problem (Cadwell and Coscoy, 2005, Wang et al., 2009). The E3 ligase responsible however, remained elusive. An interaction between US11 and the HRD1 retrotranslocation component SEL1L led to suggestions that HRD1 might be the host E3 ligase for US11 (Mueller et al., 2006). However, neither HRD1 depletion nor expression of a HRD1 dominant-negative mutant rescued US11-induced MHC-I degradation. We were puzzled that our siRNA ubiquitome screen which found TRC8 as the E3 ligase responsible for US2-mediated MHC-I dislocation, only identified the E2 ubiquitin conjugase (Ube2J2) essential for US11 activity, but failed to identify any E3 ligase (van den Boomen et al., 2014).

Subsequent progress toward the identification of the host E3 ligase responsible for US11-induced degradation came from two related genetic approaches. We performed a forward genetic screen in the near-haploid human KBM7 cell line expressing US11 and identified an uncharacterised gene TMEM129 as both essential and rate-limiting for US11-mediated MHC-I degradation (van den Boomen et al., 2014), while a related study identified TMEM129 in a genome wide shRNA screen for US11-induced degradation (van de Weijer et al., 2014). TMEM129 is an ER-resident membrane protein conserved from humans to the single cell organism *Capsaspora owczarzaki*, with no orthologue in yeast. Its cytoplasmic C-terminus contains a unique and highly conserved cysteine-rich region with similarity to the RING domain of E3 ubiquitin ligases. The classical ubiquitin RING domain consists of interspersed cysteine and histidine residues in a cross-brace motif that coordinates two zinc atoms and forms a binding platform for the E2 ubiquitin conjugase. The putative RING of TMEM129 is unusual as it predicts elongated loops not seen in other RING domains and has only cysteine residues, but no histidine for zinc coordination. From the bioinformatics standpoint it was never classified as a putative ubiquitin E3 ligase ‘RING’, which explains why we were unable to find it in our initial siRNA ubiquitome screen of predicted E3 ligases.

*In vitro* we showed the TMEM129 RING has autoubiquitination activity, the hallmark of ubiquitin RINGs and TMEM129-deficient cells show a loss of US11-induced MHC-I ubiquitination as well as retrotranslocation and proteasomal degradation (van den Boomen et al., 2014). TMEM129 is therefore a *bona fide* RING E3 ligase and the central component of US11-induced MHC-I degradation (Fig 1C). Interestingly, whereas depletion of TRC8 in the US2 system allows MHC-I to escape to the cell surface, in the absence of TMEM129, MHC-I accumulates in the ER of US11+ cells. US11 therefore not only acts as degradation factor for MHC-I but also as an ER retention factor, as originally seen with a US11 Q192L mutant (Lilley et al., 2003). We found that TMEM129 is recruited to US11 via the rhomboid pseudo-protease Derlin-1, which bridges US11 to TMEM129 and binds US11 via the polar glutamine residue 192 (Q192) in the US11 transmembrane domain and TMEM129 via its transmembrane region (Lilley and Ploegh, 2004, van den Boomen et al., 2014). Like TMEM129-deficient cells, Derlin-1-deficient cells fail to assemble a functional US11-TMEM129 degradation complex and lack US11-induced MHC-I degradation. Studies of US11-mediated degradation have therefore not only yielded insight into the role of ubiquitination in the degradation of membrane proteins from the ER, but identified a novel and unique E3 ligase which functions in a previously uncharacterised Derlin-1 dependent ERAD pathway.

## 6 The short tail of US11 allows its escape from TMEM129-mediated degradation

US11 combines both ER retention and degradation functions. This dual function allows US11 to prevent MHC-I surface expression during times when the client load is high and the TMEM129 E3 ligase is limiting, as might occur during early HCMV infection when MHC-I expression is elevated by interferon signalling. The strong interaction between MHC-I and US11 retains MHC-I in the ER but poses a potential threat to US11, which must avoid self-destruction via TMEM129. Co-degradation seemed unlikely as the half-life of US11 of ∼1 h is considerably longer than the very short half-life of US11-associated MHC-I (1–5 min). To avoid TMEM129-induced US11 degradation, US11 has a short cytoplasmic tail largely devoid of ubiquitin acceptor residues, which contrasts with the longer, lysine-rich MHC-I tail. A tail swap between US11 and MHC-I reverses their fate, inducing a rapid TMEM129-dependent degradation of US11, leaving MHC-I completely unaffected (van den Boomen et al., 2014). US11 may therefore act as a ‘pseudo-substrate’; it recruits the E3 ligase but deflects ubiquitination onto its MHC-I client. The subsequent degradation of MHC-I frees US11 to bind the next MHC-I molecule and restarts the degradation cycle. Avoiding self-degradation, either through a short cytosolic tail, or a tail devoid of ubiquitin acceptor residues might be a common feature for viral immune evasins that directly or indirectly interact with an E3 ligase. Such a strategy includes the viral proteins US2 and US3, and might extend to select host ERAD factors including Herp and SEL1L.

## 7 An auto-regulatory control loop fine tunes US11 activity and allows it to buffer changes in MHC-I expression

Although US11 avoids ubiquitination by TMEM129, in the absence of MHC-I it is itself unstable and degraded by the classical ERAD E3 ligase HRD1, which is also part of the US11 complex. Depletion of HRD1 or its retrotranslocation component SEL1L increases US11 expression without affecting MHC-I degradation. There are therefore two ongoing degradation processes within the US11 complex—the rapid degradation of MHC-I by TMEM129 and the slow turnover of US11 by the HRD1/SEL1L complex (Fig 1C). This observation explains a conundrum of US11-mediated degradation, that US11 binds SEL1L, while the TMEM129 E3 ligase does not. Why might this complex arrangement have evolved?

The HRD1/SEL1L complex has a well-defined function in the turnover of misassembled complexes of membrane proteins, including misassembled TCRα and CD3δ, and the turn-over of US11 must be seen within this context. US11 binds MHC-I in a stable complex as seen in the ER retention of MHC-I in TMEM129-deficient cells. Following the TMEM129-dependent degradation of MHC-I, we propose unpaired or ‘free’ US11 either re-binds a fresh MHC-I or exposes a degron that overlaps with the MHC binding site. Unpaired US11 is inherently unstable and the US11 degron is recognized by SEL1L, leading to HRD1-dependent degradation. In support of this model, US11 expression levels are markedly increased following expression of a soluble MHC-I which is not a substrate for TMEM129 (van den Boomen et al., 2014). Binding of US11 by soluble MHC-I masks its degron, increasing its stability. Similarly TMEM129 depletion leads to a marked increase in US11 expression. This is not because US11 is a TMEM129 substrate but because the MHC-I is no longer degraded and therefore binds US11, resulting in a stable US11-MHC-I complex. In this way, the degradation of US11 provides an autoregulatory control loop, which buffers US11 against the amount of MHC-I present in the cell. When a large amount of MHC-I is present, it will bind US11, and when MHC-I levels decrease, free US11 is degraded by HRD1 (Fig 1C).

Therefore this HRD1 mediated degradation of US11 fine-tunes US11-mediated degradation, allowing US11 to respond to changes in MHC-I concentration in the ER. If MHC-I levels increase, for example due to interferon signalling, US11 levels will concomitantly increase and instantly retain and destroy the MHC-I. Conversely when MHC-I levels are low, US11 expression will decrease. This prevents an accumulation of unnecessary ‘free’ US11 in the ER and increases the probability of TMEM129 to find US11-MHC-I complexes and induce MHC-I degradation. This fine-tuning of MHC-I degradation by targeted degradation of ‘free’ US11 is reminiscent of ‘ERAD tuning’, a process in which components of the ERAD machinery such as HERP and EDEM1 are degraded or removed in the absence of protein substrates (Bernasconi et al., 2013, Cali et al., 2008). Viral induced degradation therefore resembles host cell ERAD in several aspects.

## 8 The US2/TRC8 complex acts as a multifunctional viral degradation hub

The hijacking of host ERAD E3 ligases by HCMV US2 and US11 provides HCMV with a powerful mechanism to deplete the host cell of a key molecule essential for immune surveillance. The same strategy could be used by HCMV to degrade additional immune receptors. US2 degrades components of the MHC-II complex as well as the MHC-like molecule HFE (Ben-Arieh et al., 2001, Tomazin et al., 1999). To identify additional US2/11 substrates we took an unbiased proteomic profiling approach we term ‘plasma membrane profiling’ (Hsu et al., 2015). While US11 is remarkably MHC-I specific, analysis of US2-expressing cells suggested the degradation of MHC-like molecules is only the tip of the iceberg. It turns out US2 targets a host of unrelated cell surface proteins including a wide range of α integrins, the anti-coagulation factor thrombomodulin, the IL-12 receptor β1 chain and the NK cell ligand CD112 (Fig 1A). Degradation of all these substrates proceeds via the same TRC8 pathway as defined for MHC-I. While the US2/TRC8 complex alone is able to degrade the majority of substrates, we also uncovered an interesting example of cooperation between two HCMV viral proteins in the degradation of the NK cell ligand CD112 which required both US2 and HCMV UL141. UL141 retains CD112 in the ER from where it is targeted for degradation by the US2/TRC8 complex (Fig 1A) (Hsu et al., 2015, Prod’homme et al., 2010).

Through its appropriation of TRC8, US2 therefore functions as a virus-encoded degradation hub. US2's ability to target a variety of structurally distinct substrates makes it a highly adaptable degradation factor whose substrate specificity can be altered by the expression of different retention factors. Whereas immediate early expression of US3, for instance, enables US2 to initially focus on MHC-I degradation and avoid early T-cell recognition, late expression of UL141 might switch substrate specificity toward CD112 to prevent NK cell killing—an essential function once MHC levels are decreased. Continuous degradation of α integrins might meanwhile prevent infected dendritic cells from moving to the draining lymph node during virus reactivation. US2 thus forms a multifunctional and multifactorial viral degradation hub that hijacks mammalian TRC8-dependent ERAD to target multiple angles of anti-viral immunity.

## 9 HRD1 mediates endogenous quality control of misassembled MHC-I heavy chains

Under normal physiological conditions, in the absence of viral infection, the MHC-I heterotrimer undergoes a stringent quality control, and those molecules which fail to attain their native state are degraded via ERAD. Somewhat surprisingly, neither TRC8 nor TMEM129 are used to target misfolded MHC-I for degradation. In an independent siRNA screen, we identified the HRD1-SEL1L E3 ligase complex and the E2 Ube2J1 as required for degradation of misfolded MHC-I (Burr et al., 2011). Early recognition of the misfolded MHC-I heavy chain is mediated by the ER mannosidase EDEM1 and lectins OS9 and XTP3B (Burr et al., 2013). US2 and US11 do not therefore accelerate an endogenous pathway for MHC-I clearance but function as MHC-I-specific recruitment factors that by-pass early recognition steps within the ERAD process and force-feed a different ligase complex with ‘folding-competent’ MHC-I. However, HRD1-mediated degradation of MHC-I does share some characteristics with the US2 and US11 pathways. Like US11, HRD1 can for example degrade a lysine-less MHC-I molecule, and like US2 this degradation process does not require a cytoplasmic tail (Burr et al., 2013, Furman et al., 2003, Hassink et al., 2006). Indeed, mapping of MHC-I ubiquitination showed preferential HRD1-dependent ubiquitination on lysine residues of the MHC-I luminal domain (Burr et al., 2013).

The study of MHC-I degradation by viral and endogenous proteins has therefore identified three independent branches of mammalian ERAD, each with its characteristic E3 ubiquitin ligase, which emphasises the complexities of this important quality control pathway and provided mechanistic insight into the ongoing battle between viral infection and immune detection.
